# Protecting Low-Income Consumers in the Era of Digital Grocery Shopping: Implications for WIC Online Ordering

**DOI:** 10.3390/nu15020390

**Published:** 2023-01-12

**Authors:** Qi Zhang, Priyanka Patel, Caitlin M. Lowery

**Affiliations:** 1 School of Community and Environmental Health, Old Dominion University, Norfolk, VA 23529, USA; 2 Department of Nutrition, Gillings School of Global Public Health, University of North Carolina at Chapel Hill, Chapel Hill, NC 27599, USA

**Keywords:** WIC, online ordering, laws, regulations, consumer protection

## Abstract

The Special Supplemental Nutrition Program for Women, Infants, and Children (WIC) is now expected to allow participants to redeem their food benefits online, i.e., via online ordering, rather than only in-store. However, it is unclear how this new benefit redemption model may impact participants’ welfare since vendors may have an asymmetric information advantage compared with WIC customers. The WIC online ordering environment may also change the landscape for WIC vendors, which will eventually affect WIC participants. To protect WIC consumers’ rights in the new online ordering model, policymakers need an appropriate legal and regulatory framework. This narrative review provides that framework by reviewing the literature, legal treatises, and reports on enforceable laws and regulations in the U.S. relevant to digital marketing. The results identify key issues that may arise with adopting WIC online ordering. This paper suggests “privacy, transparency, and fairness” as guiding principles to protect the welfare of WIC participants in WIC online ordering.

## 1. Introduction

The Special Supplemental Nutrition Program for Women, Infants, and Children (WIC) is the third-largest federal nutrition assistance program in the U.S., serving approximately 6.2 million low-income infants, and children under age 5, as well as pregnant, breastfeeding, and postpartum women in the 2020 fiscal year [1]. The U.S. Department of Agriculture (USDA) provides WIC grants to 89 agencies, which include 50 states, 33 Indian Tribal Organizations, the District of Columbia, and five U.S. territories. The WIC program prescribes limited quantities of up to 10 categories of nutritious foods to child and female participants, including milk, breakfast cereal, cheese, eggs, whole-wheat bread, fish, legumes, and peanut butter, as well as cash value benefits to purchase fruits and vegetables. For infants, WIC prescribes up to four food categories, including formula, infant cereal, infant fruits and vegetables, and infant meat [1]. For each category, the quantity (or dollar amount) allowed is loaded onto electronic benefit transfer (EBT) cards, which function in a similar way to debit cards. The participants can use the EBT cards to pay for eligible foods in WIC-authorized stores across the country. Under the USDA’s general guidelines, each agency has the authority to select the eligible brands and package sizes for each food category. Therefore, WIC is a federally funded but state-operated program with complicated operation rules. More details about the program can be found in other documents [2,3].

Although online grocery shopping has increased significantly among the general US population since 2015, especially during the COVID-19 pandemic, WIC participants were not allowed to redeem their food benefits online due to WIC regulations that require a cashier’s presence for redemption [4,5]. However, the American Rescue Plan Act of 2021 granted waivers to state agencies requesting to conduct pilot online ordering and transaction projects [6]. To ensure that WIC customers have equally equitable access to online grocery shopping as other customers, the Consolidated Appropriations Act of 2021 required the USDA to establish a task force to “study measures to streamline the redemption of supplemental foods benefits that promote convenience, safety, and equitable access to supplemental foods” [7]. After evaluating alternative methods of WIC redemption, the task force strongly recommended developing rules that allow modern and intelligent ordering and purchasing methods consistent with the existing commercial model. Following the task force’s recommendation, the USDA plans to publish interim rules soon to allow WIC participants to redeem their benefits online [8].

WIC online ordering essentially provides a new grocery shopping environment for WIC participants, who may benefit from it just as other consumers benefit from online shopping. Qualitative studies show a general interest in and acceptance of WIC online ordering among the participants [9,10], and 52.6% of WIC participants in a multi-state survey stated that the unavailability of WIC online shopping was the reason they did not redeem all of their WIC benefits [11]. However, barriers still exist for state WIC agencies to implement WIC online ordering [12]. In a pilot project, only 5% of the WIC customers adopted it [13]. Therefore, how to implement WIC online ordering and improve the experience remains a challenging question for policymakers.

WIC online ordering customers are potentially vulnerable to a variety of risks. First, the food industry has developed mature online technologies, e.g., algorithms, that can target customers and influence their food choices with tailored information, such as advertisements [14,15,16]. A higher profit margin from unhealthy food choices can drive vendors to promote more unhealthy food choices to WIC participants than healthy ones [17]. Hypothetically, although WIC food benefits are restricted to only nutritious foods, under the influence of such targeted advertisements, WIC participants could be more likely to purchase non-WIC food items, e.g., unhealthy snacks, using other payment methods, such as the Supplemental Nutrition Assistance Program (SNAP) electronic benefit transfer (EBT) card or their own debit or credit cards. The SNAP program is the largest nutrition assistance program in the U.S. Different from the WIC program, SNAP loads cash-like benefits to the participants’ EBT cards so they can pay for any eligible foods in authorized stores [18]. Any individuals whose household income is at or below 130 percent of the federal poverty line are eligible for the SNAP program [19].

WIC vendors cannot be restricted from engaging in such advertising because the current WIC regulations require WIC-authorized vendors to treat WIC customers as equitably as non-WIC customers in terms of providing in-store promotions, discounts, and coupons [20]. In the online shopping environment, it is common practice for vendors to provide these targeted advertisements for healthy and unhealthy foods as “promotions” to general customers. Still, it is questionable whether the WIC customers would also be targeted in ways inconsistent with equity and WIC program goals. Therefore, it is unclear how to address these marketing concerns in the new WIC online ordering program.

Second, consumer privacy is a legitimate concern for any online transaction. Once WIC participants redeem their food benefits, the information collected by the online vendors is susceptible to identity theft or abuse, such as being shared with third parties without customers’ authorization. Consumer information can also be vulnerable to internal information abuse, even if the information is not identifiable. For example, in the current legal framework, poverty is not a protected status, unlike race/ethnicity, gender, or age. Being identified as a WIC participant, and thus flagged as low-income, could make WIC online shoppers susceptible to targeted advertising for unhealthy foods, as noted above, which could work counter to the intent of WIC to promote healthy food choices to participants.

Finally, the new WIC online ordering market may affect participants by creating business disadvantages for some current WIC vendors. The new market may benefit a few large vendors or platforms to the exclusion of smaller ones, since not every WIC-authorized vendor has the same technological capacity to develop an online ordering infrastructure for WIC redemption. WIC participants benefit from a variety of vendor choices rather than having to rely on only one or a few vendors. In theory, then, the greater capacity of larger vendors to implement online ordering could create a monopoly or oligopoly as smaller vendors do not participate in online ordering or exit from the WIC market completely, which can ultimately reduce consumer welfare. The exit of smaller vendors from the WIC program might impact WIC participants disproportionally, especially those without stable internet access or a secure location for food deliveries. Although WIC participants do not pay for WIC foods, they may pay indirectly if the costs of adopting online ordering technologies create such a market asymmetry. Therefore, how to ensure equal opportunity for all WIC-authorized vendors is important for WIC participants as well. These sorts of antitrust issues are important for policymakers to consider in the development of final rules.

Given the fast growth of online grocery shopping and the WIC policy environment, many of these issues may not be fully considered in developing the WIC online ordering program. To provide a legal framework for the new WIC initiative, this study reviews the academic and gray literature related to laws and regulations about consumer protection that may have relevant implications for WIC online ordering. Another purpose of this review is to identify the types of regulations that are appropriate to govern the operation of WIC online ordering and have the potential to prevent any negative impact on vulnerable WIC populations. By studying these secondary sources of the relevant laws and regulations, we can incorporate different views of the primary source, which are the laws and regulations themselves, into developing a new legal framework for WIC online ordering. Since the dissemination of legal studies is not limited to peer-reviewed journal publications, the study scope was extended to review the gray literature published outside of traditional academic venues. These results are timely in helping the USDA and state agencies as they develop regulations and operational guidelines to implement WIC online ordering. An ideal design will allow WIC participants to benefit from a more modern benefit redemption method while being protected from any illegal or inappropriate practices in the online shopping environment.

## 2. Methods

The narrative review of the literature covered the following target populations and key concepts:

First, the targeted populations are online consumers, including low-income women, infants, and children in a grocery shopping environment. Consumer rights were defined as the right to decide how online retailers or vendors collect, use, process, disclose, or secure personal information in that environment. Retailers were defined as merchants who sell groceries directly, e.g., Walmart, or third-party platforms which allow the merchant to sell groceries (e.g., Amazon). Groceries were defined as fresh foods or food products, e.g., infant formula. The search sources that were reviewed included literature, treatises, and reports, defined as: (1) literature is law reviews and peer-reviewed journal publications; (2) treatises are professional reviews or surveys of laws about the topic; and (3) reports are government or non-profit organizations’ overviews on the topic.

The resources to search included the following: Nexis Uni, the American Bar Association website, the Federal Trade Commission website, the Center for Digital Democracy website, the Consumer Financial Protection Bureau, and the National Consumer Law Center. The search terms included combinations of the keywords: (consumer OR client OR shopper) AND (online OR digital OR e-commerce OR web OR online) AND (food OR grocery) AND (protection OR right OR privacy OR security OR unfair OR deceptive) AND (“internet marketing” OR “digital marketing” OR “consumer data” OR “data mining” OR “web analytics” OR “online advertising” OR “targeted advertising” OR “consumer analytics” OR “targeted marketing”). The search period was from January 1, 2015, to June 20, 2022, since online grocery shopping had limited uptake in the U.S. before 2015 [4].

Moreover, since WIC is a U.S. nutrition assistance program, the location of the studies was limited to the U.S. only, and the publication had to be in English. In this review, we only included the studies that focused on the general legal framework or the federal laws, since online ordering for WIC will be regulated at the federal level. The type of publications included reviews, quantitative studies, qualitative studies, observational studies, treatises, surveys, and reports. The exclusion criteria were any publications not relevant to online shopping, any publications related to digital marketing outside of the online retail setting (such as on social media), any publications that may have had conflicts of interest with the food industry, conference proceedings or abstracts, and dissertations or theses. Two researchers independently reviewed the search results and decided whether each result met the inclusion or exclusion criteria. When there was any disagreement, a third researcher assessed and decided on inclusion or exclusion.

## 3. Results

The search yielded 13 journal articles, two reports, and one legal survey. Across these 16 resources, 11 federal laws and regulations were referenced (listed in Table 1). The themes of these laws and regulations varied from regulating electronic information to protecting target populations, which included children (Children’s Online Privacy Protection Act [COPPA]) [21], computer users (Computer Fraud and Abuse Act [CFAA]) [22], consumers (Consumer Review Fairness Act [CRFA] [23], Controlling the Assault of Non-Solicited Pornography and Marketing [CAN-SPAM] Act [24]), individuals interacting with government agencies (Electronic Communication Privacy Act [ECPA] [25] and Privacy Act [26]) and institution collection personal information (Gramm–Leach–Bliley Act (GLBA)/the Financial Services Modernization Act) [27].

The number and variety of these policies reflect the fact that the U.S. does not have a comprehensive law to protect individuals’ privacy, even though the digital market or e-commerce industry is a large segment of the U.S. economy, with a sales revenue of $578.5 billion in 2019 [33]. This patchwork system of privacy protection laws will govern WIC online ordering since they determine how businesses collect, store, and use WIC participants’ information. In particular, the Federal Trade Commission (FTC) Act and the U.S. Safe Web Act [28,29], which amends the FTC Act, provide comprehensive protection for consumers against “unfair or deceptive acts or practices in or affecting commerce.” In addition, the Telephone Consumer Protection Act (TCPA) regulates telemarketing businesses that could potentially target WIC consumers [32].

Although most relevant laws protect consumers, two additional laws protect the platform and small vendors in the market, which can potentially improve the downstream welfare of consumers as well. The first one is the Robinson–Patman Act of 1936 [30], which is an antitrust law, although it is not as prominent as the three pillars of the antitrust law system, i.e., the Sherman Antitrust Act of 1890 [34], the Clayton Antitrust Act of 1914 [35], and the FTC Act [28]. The Robinson–Patman Act of 1936 protects small businesses from the aggressive competition by chain stores. It should be noted that WIC online ordering may rely on existing platforms or create new online platforms to facilitate the exchange between WIC customers, vendors, and WIC agencies. Section 230 of the Communications Decency Act of 1996 [31] protects “interactive computer services” from liability, i.e., if the merchant is to provide the platform for goods to be exchanged, that merchant should not be liable for product defects. Whether the new online WIC platforms will be susceptible to any liability charges may affect entrepreneurs’ innovation.

A summary of the included publications is listed in Table 2. The literature review covers a wide array of topics regarding the digital market, including algorithms, digital technology, consumer privacy, consumer rights, information asymmetries, antitrust, and product liabilities. The majority of the studies focus on digital technology, which can have a profound impact on consumers’ behaviors and their welfare, especially because these technologies are new and are thus gray areas under the existing regulations (i.e., regulations might not exist for emerging technologies). The consensus of the literature is that there is no single piece of legislation that clearly governs all practices in an online shopping environment. Instead, multiple related laws and rules regulate the behaviors of vendors to protect consumers’ rights from multiple perspectives. The following sections discuss the specific topics identified by the review and their relevance to WIC online ordering.

### 3.1. Algorithm Usage in the Digital Market

An algorithm is a set of automatic rules or procedures that produce a range of outcomes given the input data or parameters [50]. Large retailers have already implemented sophisticated algorithms to personalize product recommendations for online customers [51]. Government agencies have also adopted algorithms to assist decision-making in criminal justice, education, and public assistance programs [52]. For example, the Rapid Safety Feedback Program (RSFP) used an algorithm to predict whether a missing child was killed or severely injured [53]. The WIC online ordering programs may use algorithms to determine the food benefits for which WIC consumers are eligible. Vendors that provide WIC online ordering may also have internal algorithms to optimize their operations. However, this review indicates that using algorithms can raise potential issues that need to be resolved from the regulatory perspective.

First, although machines automatically execute algorithms, they are programmed by humans. Therefore, human beings’ underlying biases or other errors may be introduced into the algorithm [49]. One prominent example is when Amazon started a same-day delivery service in Boston but excluded low-income and minority-concentrated zip codes from the service [54]. The underlying reason is that the algorithm relied on the number of Amazon Prime members in the zip code, and Prime membership served as a proxy for socio-economic status. Although Amazon eventually corrected this error, similar biases or errors in algorithms can certainly be repeated. Providing equal treatment to WIC and non-WIC customers is an essential principle in WIC operations. For instance, if WIC online ordering permits home delivery, participants should receive equal delivery services as non-WIC customers, such as the delivery time and location. How to avoid or limit these sorts of underlying biases is worth discussing [20].

Second, extensive algorithm usage can selectively empower certain merchants in the market, which could create unfair competition, and the current antitrust laws may not be able to tackle these extensive algorithms [39,45]. Merchants use algorithms to influence consumers’ online shopping behaviors, such as personalized product recommendations or targeted advertising. A particular example is Amazon. Traditionally, Amazon was treated as a third-party market. However, Amazon also sells private-label branded products, such as Amazon Basics, Presto, or Lark & Ro [45]. Amazon has developed sophisticated algorithms, such as “A9,” to promote private-label products while customers shop on their site. This private label has a price advantage in the short run. However, if algorithms dominate consumers’ shopping habits, third-party products may be gradually ousted from the markets, eventually leading to price increases for consumers. Since antitrust laws focus on price, they may not apply to Amazon’s private-label case in the short run [45].

Some algorithms are developed for consumers, such as the auto-renewal of subscriptions [39]. Although these algorithms can lower the time cost of online shopping and ease the consumer’s shopping experience, they can also harm consumers and market formation in the long run by restricting opportunities to consider new options. For example, these algorithms can create bounded choices for consumers, effectively locking them into one set of products or services, which makes other products and services unlikely to be accessible. Once again, this creates a certain degree of information monopoly in the market.

Given the potential errors or biases in algorithms, it is important to keep them subject to proper review or transparency. However, Bunnell [37] reviewed the cases in which there was a dispute on the governance of these public-funded but privately developed algorithms and found that the courts consistently treated these privately developed algorithms, even when used by the public sector, as trade secrets subject to protection. Moreover, even if the codes of these complicated algorithms were available, public agencies staffed by individuals without particular technical backgrounds may not be able to understand them fully. Therefore, in the current legislative system and policy environment, public agencies can only operate with good faith that retailer algorithms are error-free and non-biased, or wait until a significantly undesirable outcome is detected to take action, e.g., Amazon’s same-day delivery.

Scholars have proposed alternative practices or regulations to resolve this dilemma. For example, Tene et al. [49] argued for differentiating between “policy-neutral algorithms” and “policy-directed algorithms.” Policy-neutral algorithms do not embed active policy decisions in the coding process, while policy-directed algorithms are intentionally designed to fulfill the agencies’ policy goals. Policy-directed algorithms may require ethical review and strict transparency. Even given the trade secret argument for non-disclosure, the conceptual framework of the algorithm, instead of the detailed codes, can be reviewed. Both Tene et al. [49] and Bunnell [37] suggested developing independent agencies, such as an Ethics Review Commission or an Algorithm Transparency Commission, that would have the technical expertise to ensure the ethical integrity of these algorithms and to promote healthy food choices among WIC participants, an important policy goal for the WIC program [55].

### 3.2. Other Digital Technology in the Online Market

Digital technology has benefited consumers in multiple ways in the online market. Search engines can significantly reduce consumers’ search costs for desirable products. Online reviews can reduce information asymmetries between consumers and merchants. Electronic communications can help resolve issues in the purchase process and post-sale phases. Although these innovations have aided consumers in dealing with online businesses, additional regulation is necessary to address asymmetries in the digital market further [41].

The FTC and the Center for Digital Democracy (CDD) published reports to comprehensively review digital technologies in marketing and how these technologies may affect consumer welfare, especially among children [14,15]. Both reports highlighted big data analytics, which can be a powerful tool for businesses to target certain groups of consumers for marketing purposes. Low-income groups can be particularly vulnerable to these digital technologies because the latter can promote predatory products or services, such as online college degrees and subprime loans [56]. Children are also frequently targeted by unhealthy food marketing, which can have severe and long-lasting consequences on their health status, such as childhood obesity [14,57].

The United Nations Convention on the Rights of the Child (CRC) has particular clauses to protect children’s freedom from exploitation [48]: Articles 12, 32, and 36 protect children’s privacy and rights from being exploited or otherwise disadvantaged. Although the United States remains the only country that has not ratified the CRC [58] due to concerns regarding governmental interference in family lives [59], policymakers can develop regulations specifically to protect children from targeted advertising that can have negative health impacts on them.

### 3.3. Consumer Privacy

Three publications discussed consumer privacy issues in digital markets [14,40,42]. Jolly [42] pointed out the fragmented nature of the U.S. privacy law system and provided a comprehensive review of applicable laws that may protect consumers’ personal information in the digital age. The Children’s Online Privacy Protection Act (COPPA) protects children’s privacy to some degree, e.g., parental permission is required before any digital entities collect children’s (younger than 13) personal information, their data must be protected, and the use to which it may be put must be adequately disclosed [21]. However, these clauses do not necessarily protect children from targeted advertising [14]. In the WIC online ordering market, online vendors may only need general indicators of the consumers’ family status, such as having a non-breastfeeding infant, to implement a personalized advertising campaign. Therefore, the privacy domain may need to be broadened to protect WIC participants in an online ordering environment.

Gilman [40] suggests that the U.S. should adopt the five principles of the European Union’s General Data Protection Regulation (GDPR) regarding privacy: (1) transparency about any automated decision-making; (2) decisions to not be completely determined by automated profiling; (3) the right to reject the use of certain kinds of information by other entities, e.g., information on poverty status, which is not a protected class of information and knowledge of which could enable businesses to target low-income groups with exploitative ads; (4) the right for public participation in programming that is relevant to their welfare; and (5) having implementation and enforcement agents and systems, such as the FTC. These five principles may be useful in guiding the development of WIC online ordering rules. As one example, WIC stakeholders or proxies could be involved in the programming of applications used for WIC online ordering.

Beyond the specific statutes of privacy laws, the U.S. courts uphold the “notice and consent” approach to allow businesses to collect and use personal data [60]. For example, if a website notifies the users about the privacy rule and receives consent, it can use the data for business operations. Therefore, online customers bear the burden of reading and understanding these lengthy and usually incomprehensible notices, which people rarely do [61]. Vendors can use this to collect and use customer information to, for instance, develop algorithms to target particular customers for marketing or advertising purposes. Moreover, suppose the customer’s information can be considered to have been voluntarily disclosed to a third party. In that case, a Supreme Court ruling voids the protection of customer privacy under the Fourth Amendment (the “third-party” doctrine) [62]. This “notice and consent” approach disproportionately disadvantages low-income populations because many low-income people have levels of education below what would be required to understand what they agree to under vendors’ notices and disclosures [40]. Therefore, a separate entity with the knowledge to understand these disclosures may be needed to represent consumers as supervisors of the implementation and enforcement of these privacy rules in the online market.

### 3.4. Information Asymmetries

Information asymmetries exist in the digital market, as in other markets; sellers usually have more information about the product or services than consumers [63]. Mandatory disclosure is intended to address such asymmetries. However, as noted above, these types of disclosures are not necessarily effective since they often contain complex information presented in legal rather than plain language, which tends to overwhelm consumers rather than resolve the information asymmetries [46].

At the same time, the digital market provides powerful tools for consumers to address some information asymmetries. For example, consumers can use search engines to examine the product features, such as price or size, before the purchase. Moreover, online reviews allow consumers to share their post-purchase experiences about the product or service [38]. However, information asymmetries cannot be eliminated in the digital market. Shchory [46] argues that certain qualities, namely credence qualities, cannot be verified by consumers even after the purchase, e.g., the accuracy of the nutrition label or the probability of a product malfunction. Traditional consumer protection laws rely on three main approaches: mandatory disclosure, no unfair or deceptive acts, and setting quality standards, which public agencies enforce. However, all these three approaches may not address the information asymmetries on credence qualities, which requires innovative ways to protect consumers. Shchory [46] suggested that to address the digital market asymmetry issues, regulators can set up some centralized public portals, such as Wikipedia, to allow information sharing from varying sources.

Moreover, Dammann [38] pointed out the significant limitations of consumer reviews in addressing the information asymmetries in digital markets. Although the Consumer Review Fairness Act of 2016 prohibits banning consumer reviews in contractual clauses, fake reviews, biases toward early buyers’ reviews, and other psychological or behavioral biases significantly prevent online reviews from fully protecting consumers. Therefore, consumer reviews cannot replace legislation or regulations to address the information asymmetries in the online market.

### 3.5. Platform Liabilities

Platforms emerge in the digital economy to provide a venue, often critical, for various vendors and consumers to conduct transactions. Existing platforms, e.g., Instacart, or newly developed platforms, may be adapted to facilitate WIC online ordering. However, it may still be ambiguous as to whether these platforms have immunity from liability [36,44]. Answers to this question are important for consumers and vendors since over-regulation, i.e., making platforms liable for products or services they should not be liable for, may stifle innovation in a newly developed market. However, without proper regulations, vulnerable consumers, such as WIC consumers, may be harmed by the negligence of the platform, e.g., ranking near-expiration food items higher for WIC customers. Therefore, it is important to examine platform liabilities within the existing legislative framework.

So far, court rulings have favored platform developers such as Amazon. Multiple courts have ruled that digital platforms were not liable for the deficiency of the products or services exchanged on the platform [36]. Section 230 of the Communications Decency Act (CDA) provides immunity to “interactive computer services” but not to “information content providers” [44], and most platforms, such as Uber or Amazon, were classified as belonging to service providers instead of content providers. However, both Bullard [36] and McPeak [44] argued that emerging technologies might make these binary classifications outdated, and legislative reform may be needed. For example, Amazon has developed comprehensive services for vendors, e.g., fulfillment by Amazon, that streamlines the selling process from packaging, warehousing, and delivery, all the way through to customer service [64]. Therefore, scholars suggest that Amazon should not be ruled as a mere information provider, like a newspaper, immune to liabilities [36]. Instead, McPeak [44] argued that joint enterprise liability in tort law might be applicable in this environment. Moreover, the binary classification of “interactive service” and “content provider” may be outdated, given the current level and extent of technology innovation. Both scholars call for the evolution of the current tort laws to extend strict product liabilities to the platform [36,44].

### 3.6. Antitrust

The fundamental principle of antitrust law is to protect consumers by creating the conditions for fair pricing through vendor competition since monopoly or oligopoly conditions can drive prices up. However, due to the disparity in access to technology and resources across vendors, some vendors may have a disproportionate advantage in the online market, such as WIC online ordering. In contrast, other vendors may not be able to enter the online market. For example, the SNAP Online Purchasing Pilot includes large, national, or regional chain grocery stores but rarely any small convenience stores [65]. Therefore, it is crucial to understand the legal implications of this limited competition in the special online markets.

The current antitrust laws protect competition, not competitors, and use price as the primary measurement to evaluate the competition [43]. Therefore, small vendors may not claim antitrust protection if other vendors, such as Amazon, can provide a lower wholesale price to the consumers. However, Kim [43] argued for a novel application of the Robinson–Patman Act (RPA) of 1936, since the RPA was originally drafted to protect small businesses against aggressive chain stores, although the courts may not view the application the same way as she argued. The antitrust laws are not necessarily applied to only large vendors in the digital market. For example, some small vendors, who do not have significant market power, may nevertheless manipulate information to get a “free ride” on the marketing power of larger infomediaries, e.g., by faking Google reviews to gain an unfair advantage against other competitors. Shchory and Gal (2021) [47] called these competitors “market power parasites,” which may not be the direct target of the existing antitrust laws, the FTC Act, and other consumer protection laws. Due to unequal technological advantages across WIC vendors, some small vendors may leverage these “parasite practices” to balance their competitive disadvantage against large vendors in the WIC online ordering market. To address the threats of these market power parasites, the “fraud-on-the-market” rule of securities laws can be borrowed to form the framework of a “fraud-on-the-online-information-market” law [47]. Limiting unfair competition in the online market may eventually provide consumers with lower prices and higher-quality products and services.

In summary, the literature review indicated that digital technologies in the online market could threaten consumers’ privacy and welfare through unfair competition. No comprehensive laws are available to regulate all the issues in the online market. Instead, regulation is evolving alongside emerging technologies and practices, indicating that there is room for and a need to consider consumer protection regulation in the age of online shopping, especially when it pertains to consumers participating in federal food and nutrition assistance programs.

## 4. Discussion

WIC online ordering will be a new method for participants to redeem their food benefits. This review of the relevant literature generates important implications for how policymakers might develop proper regulations or guidelines to protect WIC consumers in this new redemption environment. Based on the review results, we propose the principles of privacy, transparency, and fairness for WIC online ordering programs.

Privacy. First, we recommend that stakeholders develop regulations to ensure that WIC customers’ private information is properly collected, used, and protected. Online marketplaces or vendors must collect WIC customers’ information for identity verification. However, it is yet to be determined what constitutes the minimum required information to be collected for online redemption. Second, WIC customers are vulnerable low-income populations that include infants and children, making it essential to protect their participation status and identities. Third, compared with regular online shoppers, WIC participation status is unique. It can be abused for targeted marketing or advertising purposes, i.e., the vendors may “discriminate” against the consumers based upon their WIC participation status or low-income status, since poverty is not a protected class in the current legislation. Like other customers, participants’ identifiable information should be securely stored and protected from unethical use by vendors or unauthorized third-party access. However, since under-redemption is prevalent among WIC participants, it is not clear whether the vendors or the marketplace should be allowed to leverage participation status and contact information (e.g., email or phone numbers) for healthy food promotion, e.g., for WIC benefit redemption.

One possible solution is to establish a “firewall” between the WIC online ordering customers’ information and other online shopping customers’ information, with no information crossing through the firewall. For example, vendors can promote redemptions of eligible WIC foods by using participants’ redemption records and contact information but cannot promote non-WIC-eligible infant products to the WIC participants if these products are not promoted to other non-WIC customers at the same time, thus ensuring equal treatment for WIC and non-WIC customers.

Transparency. Second, any algorithms, data collection, or other digital technologies used by online vendors or platforms must be transparent to WIC customers, WIC agencies, and/or their proxies. Some WIC customers may not have the education needed to understand legal language and, as parents, they may have limited time to review complex privacy policies. How to properly present all disclosure documents and notifications at an appropriate level of simplicity to obtain participants’ informed decisions about the use of their personal information needs more clear guidance. Otherwise, information overload may put WIC customers in a vulnerable situation during the consent process.

Moreover, any program operation in WIC online ordering should be transparent to federal and state WIC agencies. These agencies can ensure that errors or biases are not introduced into the algorithms or digital operations used in the program. However, algorithms and other digital technologies can be complicated for non-experts to evaluate, including the WIC agencies. Furthermore, disclosure may encounter pushback from the vendors in the name of protecting their trade secrets. To strike a balance between transparency requirements and trade secret protections, one possible solution would be to develop an independent “transparency committee” consisting of experts in digital technology and WIC operations to review the algorithms and internal operations, with the members being prohibited from disclosing the technical details to other parties [37,49].

Fairness. Finally, the fairness principle is embedded in the FTC Act to protect consumers. In the WIC online ordering program, this principle can be implemented differently. First, online vendors should treat WIC consumers like other online grocery shopping consumers regarding services, e.g., delivery. This is required under the current federal regulation, and the implementation of this principle needs to be carefully supervised and enforced. Vendors should not repeat the same mistake Amazon made by not covering the zip codes having a high concentration of low-income minorities [54]. Second, online vendors should be allowed to charge WIC agencies fair prices for food products redeemed online. WIC agencies usually contract with vendors for eligible food benefits at a peer-group price [66]. However, the prices of online groceries can vary more significantly across time, locations, and brand names than offline stores [67]. More importantly, online vendors often apply pricing algorithms that personalize prices based on multiple factors, such as zip code and competitors’ prices. Additional regulation may be needed to ensure that the WIC agencies receive fair prices for food products redeemed online. How to set a fair cost for the redeemed benefits needs further exploration among the vendors, the platforms, and the WIC agencies.

Another major issue is ensuring fair competition in the online environment so that WIC customers’ welfare will not be negatively impacted. SNAP launched its online purchasing pilot program in 2014. Although the program has expanded significantly in the last few years, only a few grocery stores accept SNAP benefits online, most of which are national or regional chain grocery stores [66]. A significant number of WIC-authorized vendors are small or medium-sized grocery stores. Moreover, WIC has a special type of vendor, A50 stores or often called “WIC-only” stores, which carry foods that are all or almost all WIC-eligible; they receive more than half of their revenue from the WIC program. It is unclear how WIC online ordering would affect small or medium-sized stores like these. Suppose WIC customers become more used to online ordering than in-store redemptions. In that case, these smaller stores may no longer participate in the WIC program, limiting the choices for the redemption of benefits for WIC customers in neighborhoods where food access is limited. For example, rural households in the SNAP online purchasing pilot program had significantly less vendor coverage than urban households [68]. Since the fundamental principle of the antitrust laws is to protect the competition but not the competitors, large online vendors can lower the food costs for WIC agencies in the short term, which may not trigger the antitrust laws. However, a monopoly or oligopoly in WIC online ordering could occur. It may eventually affect offline WIC customers’ welfare if these small or medium-sized stores, like A50 stores, quit the WIC market totally. It is worth considering how to define fair competition in the WIC online ordering market, with WIC consumers’ welfare at the center.

In summary, the privacy, transparency, and fairness principles can be applied to multiple aspects of the new WIC online ordering programs. However, the implementation details still require coordinated efforts between multiple stakeholders in the program.

## 5. Conclusions

With the introduction of WIC online ordering, multiple legal and regulatory issues have arisen. This review discusses ways WIC customers may be vulnerable in the new digital environment. Considering the applicable laws can help policymakers develop regulations that protect WIC customers’ welfare in this new online ordering era.

## Figures and Tables

**Table 1:** Applicable Federal Laws and Regulations Related to WIC Online Ordering.

Title	Legislation Year	Activities Regulated	Relevancy to WIC Online Ordering
Children’s Online Privacy Protection Act (COPPA) [21]	1998	Collecting children’s (under 13 years) information online	WIC infants’ and children’s information
Computer Fraud and Abuse Act (CFAA) [22]	1986	Computer security	WIC participants’ information
Consumer Review Fairness Act (CRFA) [23]	2016	Online consumer reviews are not banned in contractual clauses	WIC customers should be allowed to post online consumer reviews for WIC online ordering
Controlling the Assault of Non-Solicited Pornography and Marketing (CAN-SPAM) Act [24]	2003	Commercial email	Email marketing toward WIC consumers
Electronic Communication Privacy Act (ECPA) [25]	1986	Electronic communication	Electronic communication with WIC consumers
Federal Trade Commission Act [28]/U.S. Safe Web Act [29]	1914 and 2006	“Unfair or deceptive acts or practices in or affecting commerce”	WIC consumers’ choices
Gramm–Leach–Bliley Act (GLBA)/the Financial Services Modernization Act [27]	1999	Personal information collected and/or shared by financial institutions	WIC participants’ information
Privacy Act [26]	1974	Regulate the government agencies on how to collect and store individuals’ information	Protecting WIC participants’ online ordering data
Robinson–Patman Act [30]	1936	Protect small businesses from aggressive competition within chain stores	Protecting small WIC-authorized vendors from Amazon
Section 230 of the Communications Decency Act [31]	1996	Protecting platforms that provide “interactive computer services” from liability	Not necessarily applicable to platforms that provide WIC online ordering
Telephone Consumer Protection Act (TCPA) [32]	1991	Telemarketing	Telemarketing toward WIC consumers

**Table 2:** Summary Results of the Included Studies That are Relevant to WIC Online Ordering.

Number	First Author’s Last Name	Year	Publication Type	Theme	Main Argument	Implications for WIC Online Ordering
1	Bullard [36]	2019	Journal Article	Platform liability	Although recent court rulings relieved Amazon from strict liability for defective products, Amazon can still be potentially responsible through certain third-party arrangements, such as fulfillment by Amazon. Tort law can evolve through the modernization of e-commerce.	Online vendors may adopt some terms with third-party vendor or manufacturers, such as Amazon Services Business Solutions Agreement, to indemnify the online vendor from the potential lawsuit; WIC online ordering program may consider the exemption of the online vendors, especially in the early stage of the program
2	Bunnell [37]	2020	Journal Article	Algorithm and consumer protection	Privately developed, publicly- deployed algorithms can have programming errors, inherent biases, or lack of transparency, which could harm the public. However, the courts consistently treat these algorithms as trade secrets for protection. Therefore, it is necessary to regulate these publicly operated algorithms in some way.	WIC online ordering should consider developing an independent agency to regulate the algorithm, e.g., the Algorithm Transparency Commission, with oversight including the purpose, the content, and the usage of the algorithm.
3	Chester [14]	2021	Technical Report	Digital technology and consumer protection; consumer privacy	An overview of the digital marketing and big data practices’ threat to children’s food consumption and regulation recommendations	Merchants should self-regulate to limit WIC participants’ exposure to unhealthy food advertisements while redeeming WIC benefits online
4	Dammann [38]	2020	Journal Article	Information asymmetries and consumer protection	Online consumer review is unlikely to be optimized by merchants and is insufficient to resolve the information asymmetry problem between consumers and merchants in e-commerce.	WIC online ordering should allow WIC participants to post consumer reviews, although the reviews may not fully protect WIC consumers.
5	Federal Trade Commission [15]	2016	Technical Report	Digital technology and consumer protection	Multiple federal laws protect consumers in the era of big data, including Fair Credit Reporting Act, Equal Opportunity Laws, and the Federal Trade Commission Act.	WIC online ordering regulations should be based on the applicable federal laws to ensure equal treatment of WIC customers and protect their welfare.
6	Gal [39]	2017	Journal Article	Algorithm and consumer protection	Algorithms used by consumers can ease the purchase decision process, lower the transaction and time costs, and reduce consumers’ bias. However, it can impact the market structure, especially by increasing the market share of certain merchants, which needs to be regulated by antitrust law.	The consumer algorithm may help WIC participants automatically redeem WIC benefits, increase their redemption rates, and improve retention. However, the extensive usage of algorithms may benefit vendors that apply these algorithms and reduce the market competition from other vendors.
7	Gilman [40]	2020	Journal Article	Consumer privacy	The lack of comprehensive privacy laws disproportionately harms the low-income population’s welfare. The five principles of the General Data Protection Regulation (GDPR, EU) may advance economic justice for low-income people.	WIC online ordering regulation could implement these principles, e.g., public participation, to protect WIC consumers from data privacy deprivation.
8	Helveston [41]	2016	Journal Article	Digital technology and consumer protection	Digital technology empowered consumers but cannot eliminate the vulnerability of consumers in digital marketing. Government agencies are slow to respond to the threat to consumers. Regulation in the digital market is needed.	WIC regulation may prohibit merchants from manipulating the goods and reviews in online orders. Moreover, the WIC agency may create a centralized platform or portal for WIC consumers to review the merchants, barring term discrimination and limiting the use of WIC-related data (e.g., for the internal algorithm).
9	Jolly [42]	2016	Legal Survey	Consumer privacy	There is no single, comprehensive federal law regulating data privacy and activities related to personal information.	Regulating WIC online ordering needs perspectives from multiple applicable laws, such as regulating online advertisement directed toward WIC consumers, maintaining proper electronic communications with them, and protecting private information provided by these consumers.
10	Kim [43]	2021	Journal Article	Antitrust	The Robinson–Patman Act protects small businesses from aggressive competition from large chain-stores, although courts limit the application of the RPA since consumers, instead of some competitors, are the ultimate parties protected under the antitrust laws. However, certain legal grounds are available for small businesses to apply RPA against Amazon in the digital market era.	Amazon or other large online grocery stores may have disproportionate advantages against small WIC vendors with regards to online ordering. It is worth investigating how to ensure fair competition among WIC vendors and ultimately protect WIC consumers’ welfare.
11	McPeak [44]	2021	Journal Article	Platform liability	Section 230 of the Communications Decency Act (CDA) protects platforms that provide interactive computer services from liability. However, under the joint enterprise liability framework, online marketplaces can still be liable for product deficiency.	Guidance is needed on whether online platforms that host WIC- authorized vendors online are subject to the protection of Section 230 of CDA.
12	Pankratz [45]	2020	Journal Article	Algorithm and consumer protection	Amazon or other e-commerce platforms sell their own private- label products and third-party products. The internal algorithm, e.g., Amazon’s A9, may promote their private label products more than the third-party products. Although that practice may lower consumers’ price in the short term, the long-term impact on price may be a threat to consumers’ welfare. Therefore, regulations may be needed to force Amazon or similar platforms to disclose private-label products or any advertising practices.	Amazon or other e-commerce platforms that host WIC vendors must label their private-label products clearly and ensure that other third-party vendors are not squeezed out by Amazon in the WIC online ordering market.
13	Shchory [46]	2020	Journal Article	Information asymmetries and consumer protection	Information overloading and asymmetries exist in e-commerce. The credence qualities are product qualities challenging for consumers to search for pre-purchase, or experience post-purchase. The regulators can create a public portal, such as Wikipedia, to provide an open forum for stakeholders to exchange information regarding credence qualities.	An open forum regulated by public agents may protect WIC consumers with respect to their online ordering redemptions.
14	Shchory and Gal [47]	2021	Journal Article	Antitrust	Some market players, called “market power parasites,” manipulated market information with various strategies to compete unfairly against other market players. The current antitrust laws may not be adequate to regulate the market power parasites. The fraud-on-the-market rule in securities laws may be extended to become the fraud-on-the-online- information-market rule.	WIC online ordering program rules may prohibit vendors from engaging in unfair misinformation campaigns for competitive advantages.
15	Tatlow-Golden [48]	2020	Journal Article	Digital technology and consumer protection	The data-driven digital food marketing industry threats children’s health with unhealthy food marketing. Articles 12, 32 and 36 of CRC protect children’s privacy and rights from being exploited economically or in other ways. Therefore, States should regulate digital media and marketing to enforce these rights.	Although domestic laws are vague, the WIC agencies should consider following the CRC’s principles to protect children’s privacy, e.g., banning profiling of children in WIC online ordering
16	Tene [49]	2018	Journal Article	Algorithm and consumer protection	Errors or biases in the algorithms may mirror human beings’ underlying errors or biases. A distinction could be made between policy-neutral algorithms and policy-driven algorithms. Strict transparency and ethical reviews are obligated.	WIC merchants should disclose any algorithms used in WIC online ordering to the customers and the WIC agencies. WIC agencies may consider an ethics review commission to review any possible algorithms that may impact WIC online ordering customers.

## Data Availability

Publicly available.

## References

[1] U.S. Department of Agriculture/Economic Research Service (2022). WIC Program. https://www.ers.usda.gov/topics/food-nutrition-assistance/wic-program/.

[2] Congressional Research Service A Primer on WIC: The Special Supplemental Nutrition Program for Women, Infants, and Children. Congressional Research Service. R44115. https://crsreports.congress.gov/product/pdf/R/R44115.

[3] (2013). U.S. Department of Agriculture/Food Nutrition Service WIC Laws and Regulations. https://www.fns.usda.gov/wic/wic-laws-and-regulations.

[4] Saphores J.D., Xu L. (2021). E-shopping changes and the state of e-grocery shopping in the US—Evidence from national travel and time use surveys. Res. Transp. Econ..

[5] 7 CFR § 246.12. Food Delivery Methods. https://www.law.cornell.edu/cfr/text/7/246.12.

[6] U.S. Department of Agriculture (2021). WIC Supports Online Ordering and Transactions in WIC. https://www.fns.usda.gov/wic/supports-online-ordering-transactions.

[7] U.S. Department of Agriculture (2021). Task Force on Supplemental Food Delivery in the WIC Program—Recommendations Report. https://www.fns.usda.gov/wic/food-delivery-task-force-recommendations-report.

[8] U.S. Department of Agriculture (2021). Special Supplemental Nutrition Program for Women, Infants and Children (WIC): WIC Online Ordering and Transactions. https://www.reginfo.gov/public/do/eAgendaViewRule?pubId=202104&RIN=0584-AE85.

[9] Zimmer M.C., Beaird J., Steeves E.T.A. (2021). WIC participants’ perspectives about online ordering and technology in the WIC program. J. Nutr. Educ. Behav..

[10] Zimmer M.C., McElrone M., Steeves E.T.A. (2021). Feasibility and acceptability of a “click & collect” WIC online ordering pilot. J. Acad. Nutr. Diet..

[11] Ritchie L.L.D., Felix C., Sallack L., Chauvenet C., Machell G., Whaley S. (2022). Multi-State WIC Participant Satisfaction Survey: Cash Value Benefit Increase During COVID.

[12] Nitto A.M., Calloway E.E., Anderson Steeves E.T., Wieczorek Basl A., Papa F., Kersten S.K., Hill J.L. (2022). State agencies’ perspectives on planning and preparing for WIC online ordering implementation. Nutrients.

[13] Zhang Q., Park K., Zhang J., Tang C. (2022). The online ordering behaviors among participants in the Oklahoma women, infants, and children program: A cross-sectional analysis. Int. J. Environ. Res. Public Health.

[14] Chester J., Montgomery K.C., Kopp K. (2021). Big Food, Big Tech, and the Global Childhood Obesity Pandemic.

[15] Federal Trade Commission (2016). Big Data: A Tool for Inclusion or Exclusion? Understanding the Issues. https://www.ftc.gov/system/files/documents/reports/big-data-tool-inclusion-or-exclusion-understanding-issues/160106big-data-rpt.pdf.

[16] Harris J.L., Frazier III W., Kumanyika S., Ramirez A. (2019). Increasing Disparities in Food-Related Advertising Targeted to Black Youth. https://uconnruddcenter.org/wp-content/uploads/sites/2909/2020/09/TargetedMarketingTwoPagerBlackConsumers.pdf.

[17] Bennett R., Zorbas C., Huse O., Peeters A., Cameron A.J., Sacks G., Backholer K. (2020). Prevalence of healthy and unhealthy food and beverage price promotions and their potential influence on shopper purchasing behaviour: A systematic review of the literature. Obes. Rev..

[18] U.S. Department of Agriculture Supplemental Nutrition Assistance Program. https://www.fns.usda.gov/snap/supplemental-nutrition-assistance-program.

[19] U.S. Department of Agriculture Cost of Living Adjustment (COLA) Information. https://www.fns.usda.gov/snap/supplemental-nutrition-assistance-program.

[20] U.S. Department of Agriculture (2014). Vendor Management: Incentive Items, Vendor Discounts, and Coupons. https://www.fns.usda.gov/wic/vendor-management-incentive-items-vendor-discounts-and-coupons.

[21] Federal Trade Commission (1998). Children’s Online Privacy Protection Act (COPPA). https://www.ftc.gov/legal-library/browse/rules/childrens-online-privacy-protection-rule-coppa.

[22] U.S. Department of Justice (1986). Computer Fraud and Abuse Act. https://www.justice.gov/jm/jm-9-48000-computer-fraud.

[23] U.S. Congress (2016). H.R. 5111—Consumer Review Fairness Act of 2016. https://www.congress.gov/bill/114th-congress/house-bill/5111.

[24] Federal Trade Commission (2003). Controlling the Assault of Non-Solicited Pornography and Marketing Act of 2003 (CAN-SPAM Act). https://www.ftc.gov/legal-library/browse/statutes/controlling-assault-non-solicited-pornography-marketing-act-2003-can-spam-act.

[25] U.S. Department of Justice (1986). Electronic Communications Privacy Act of 1986 (ECPA): Justice Information Sharing. https://bja.ojp.gov/program/it/privacy-civil-liberties/authorities/statutes/1285.

[26] U.S. Department of Justice (2022). Privacy Act of 1974. https://www.justice.gov/opcl/privacy-act-1974#:~:text=The%20Privacy%20Act%20of%201974%2C%20as%20amended%2C%205%20U.S.C.,of%20records%20by%20federal%20agencies.

[27] Federal Trade Commission (1999). Gramm-Leach-Bliley Act. https://www.ftc.gov/business-guidance/privacy-security/gramm-leach-bliley-act.

[28] Federal Trade Commission (1914). Federal Trade Commission Act. https://www.ftc.gov/legal-library/browse/statutes/federal-trade-commission-act.

[29] Federal Trade Commission (2006). U.S. Safe Web Act. https://www.ftc.gov/legal-library/browse/statutes/us-safe-web-act.

[30] Cornell Law School (2022). 15 U.S. Code § 13—Discrimination in price, services, or facilities. https://www.law.cornell.edu/uscode/text/15/13.

[31] Cornell Law School (2022). 47 U.S. Code § 230—Protection for private blocking and screening of offensive material. https://www.law.cornell.edu/uscode/text/47/230.

[32] Federal Communications Commission (1991). Telephone Consumer Protection Act 47 USC § 227. https://www.fcc.gov/sites/default/files/tcpa-rules.pdf.

[33] U.S. Census Bureau (2021). 2019 E-Stats Report: Measuring the Electronic Economy. https://www.census.gov/newsroom/press-releases/2021/e-estats-report-electronic-economy.html.

[34] National Archives (2022). The Sherman Antitrust Act of 1890. https://www.archives.gov/milestone-documents/sherman-anti-trust-act.

[35] Cornell Law School (2022). Clayton Antitrust Act of 1914. https://www.law.cornell.edu/wex/clayton_antitrust_act.

[36] Bullard R. (2019). Out-teching products liability: Reviving strict products liability in an age of Amazon. North Carol. J. Law Technol..

[37] Bunnell N. (2020). Remedying public-sector algorithmic harms: The case for local and state regulation via independent agency. Colum. JL Soc. Probs..

[38] Dammann J. (2020). Electronic word of mouth and consumer protection: A legal and economic analysis. S.C. Law. Rev..

[39] Gal M.S., Elkin-Koren N. (2017). Algorithmic consumers. Harv. J. L. Tech..

[40] Gilman M.E. (2020). Five privacy principles (from the GDPR) the United States should adopt to advance economic justice. Ariz. St. L.J..

[41] Helveston M.N. (2016). Regulating digital markets. N.Y.U. J. L. Bus..

[42] Jolly L. (2016). US Privacy and Data Security Law: Overview.

[43] Kim K. (2021). Amazon-induced price discrimination under the Robinson–Patman act. Colum. L. Rev..

[44] McPeak A. (2021). Platform immunity redefined. Wm. Mary L. Rev..

[45] Pankratz R. (2020). Duty to disclose: Amazon’s e-commerce platform, private-label, and the need for disclosure. Kan. JL Pub. Pol’y.

[46] Shchory N.B. (2020). Information asymmetries in e-commerce: The challenge of credence qualities. J. High Tech. L..

[47] Shchory N.B., Gal M. (2021). Market power parasites: Abusing the power of digital intermediaries to harm competition. Harv. J. L. Tech..

[48] Tatlow-Golden M., Garde A. (2020). Digital food marketing to children: Exploitation, surveillance and rights violations. Glob. Food Sec..

[49] Tene O., Polonetsky J. (2018). Taming the Golem: Challenges of ethical algorithmic decision-making. NCJL Tech..

[50] Cormen T.H., Leiserson C.E., Rivest R.L., Stein C. (2022). Introduction to Algorithms.

[51] Yuan M., Pavlidis Y., Jain M., Caster K. Walmart Online Grocery Personalization: Behavioral Insights and Basket Recommendations. Proceedings of the International Conference on Conceptual Modeling.

[52] Levy K., Chasalow K.E., Riley S. (2021). Algorithms and decision-making in the public sector. Ann. Rev. L. Soc..

[53] Gillingham P. (2019). Can predictive algorithms assist decision-making in social work with children and families?. Child Abus. Rev..

[54] Kalkanci B., Rahmani M., Toktay L.B. (2019). The role of inclusive innovation in promoting social sustainability. Prod. Oper. Manag..

[55] U.S. Department of Agriculture (2018). Special Supplemental Nutrition Program for Women, Infants, and Children (WIC). Food Package Policy and Guidance. https://wicworks.fns.usda.gov/sites/default/files/media/document/WIC%20Food%20Package%20Policy%20Guidance%20Mar2018_508c.pdf.

[56] Lubis A.N. (2018). Evaluating the customer preferences of online shopping: Demographic factors and online shop application issue. Acad. Strateg. Manag. J..

[57] Boyland E., Thivel D., Mazur A., Ring-Dimitriou S., Frelut M.L., Weghuber D. (2020). Digital food marketing to young people: A substantial public health challenge. Ann. Nutr. Metab..

[58] United Nations Office of the High Commissioner for Human Rights (2022). Ratification status for the CRC-Convention on the Rights of the Child. https://tbinternet.ohchr.org/_layouts/15/TreatyBodyExternal/Treaty.aspx?Treaty=CRC&Lang=en.

[59] Simon J., Luetzow A., Conte J.R. (2020). Thirty years of the convention on the rights of the child: Developments in child sexual abuse and exploitation. Child Abuse Negl..

[60] Reidenberg J.R., Russell N.C., Callen A.J., Qasir S., Norton T.B. (2014). Privacy harms and the effectiveness of the notice and choice framework. I/S J. L. Pol’y Info. Soc’y c.

[61] Ben-Shahar O., Schneider C.E. (2014). More Than You Wanted to Know: The Failure of Mandated Disclosure.

[62] Price M.W. (2015). Rethinking privacy: Fourth amendment papers and the third-party doctrine. J. Nat’l Sec. L. Pol’y.

[63] Mavlanova T., Benbunan-Fich R., Koufaris M. (2012). Signaling theory and information asymmetry in online commerce. Inf. Manag. J..

[64] Lai G., Liu H., Xiao W., Zhao X. (2022). “Fulfilled by amazon”: A strategic perspective of competition at the e-commerce platform. Manuf. Serv. Oper. Manag..

[65] U.S. Department of Agriculture SNAP Online Purchasing Pilot. https://www.fns.usda.gov/snap/online-purchasing-pilot.

[66] Oliveira V., Frazão E. (2009). The WIC Program: Background, Trends, and Economic Issues, 2009 Edition. Economic Research Report Number 73. https://files.eric.ed.gov/fulltext/ED508195.pdf.

[67] Aparicio D., Metzman Z., Rigobon R. (2021). The pricing strategies of online grocery retailers. NBER Work. Pap. Ser..

[68] Foster I.S., Liu S.Y., Hoffs C.T., LeBoa C., Chen A.S., Rummo P.E. (2022). Disparities in SNAP online grocery delivery and implementation: Lessons learned from California during the 2020–2021 COVID pandemic. Health Place.

